# Heterogeneity in Past Year Cigarette Smoking Quit Attempts among Latinos

**DOI:** 10.1155/2012/378165

**Published:** 2012-05-17

**Authors:** Daniel A. Gundersen, Sandra E. Echeverria, M. Jane Lewis, Gary A. Giovino, Pamela Ohman-Strickland, Cristine D. Delnevo

**Affiliations:** ^1^School of Nursing, University of Medicine & Dentistry of New Jersey, 65 Bergen Street, Room GA-225, Newark, NJ 07101-2012, USA; ^2^Department of Epidemiology, School of Public Health, University of Medicine & Dentistry of New Jersey, 683 Hoes Lane West, Piscataway*‚* NJ 08854, USA; ^3^Department of Health Education and Behavioral Science, School of Public Health, University of Medicine & Dentistry of New Jersey, 683 Hoes Lane West, Piscataway, NJ 08854, USA; ^4^Department of Community Health and Health Behavior, School of Public Health and Health Professions, University at Buffalo, The State University of New York, 310 Kimball Tower, Buffalo, NY 14214-8028, USA; ^5^Department of Biostatistics, School of Public Health, University of Medicine & Dentistry of New Jersey, 683 Hoes Lane West, Piscataway, NJ 08854, USA

## Abstract

*Objective*. Examine the association between English language proficiency (ELP) and immigrant generation and having made a cigarette smoking quit attempt in the past 12 months among Latinos. Examine if gender moderates the association between acculturation and quit attempts. *Methods*. Latino past year smokers from the 2003 and 2006/07 Tobacco Use Supplement to the Current Population Survey were analyzed. Logistic regression was used to examine the association between quit attempt and ELP and immigrant generation, controlling for demographics and smoking characteristics. *Results*. Latinos with poor ELP were more likely to have made a quit attempt compared to those with good ELP (adjusted odds ratio [AOR] = 1.22, confidence interval [CI]: 1.02–1.46) after controlling for demographic and smoking characteristics. First (AOR = 1.21, CI: 1.02–1.43) and second generation immigrants (AOR = 1.36, CI: 1.12–1.64) were more likely than third generation immigrants to have made a quit attempt in the past 12 months. *Conclusion*. Quit behaviors are shaped by differences in language ability and generational status among Latinos. This underscores the need to disaggregate Latinos beyond racial/ethnic categories to identify subgroup differences relevant for smoking and smoking cessation behaviors in this population.

## 1. Introduction

Research on cigarette smoking among Latinos has explored differences with respect to acculturation [1–14], that is, “the process by which groups or individuals integrate the social and cultural values, ideas, beliefs, and behavioral patterns of their culture of origin with those of a different culture” [15]. Acculturation has been conceptualized and measured several ways, but public health research has typically included items on language preference and proficiency and the extent of contact with coethnic members, although more recent work has challenged this limited view of acculturation processes [16]. In general, this research has demonstrated that Latinas with higher levels of acculturation are more likely to smoke than Latinas with lower levels of acculturation [1, 3, 4, 6, 8–12, 14], though two studies found no association [7, 13]. Among men, however, the evidence generally finds no association [4, 7, 9, 12–14], and among the studies where a significant association was found the results were inconsistent [6, 8, 10].

The research on patterns of cigarette smoking among Latinos has largely focused on current smoking and differences in prevalence. However, prevalence is influenced by both initiation and cessation, and it is imperative for public health to understand the patterns of these behaviors as well. In particular, calls have been made to better understand cessation behaviors among racial/ethnic minorities [17, 18]. While some research has examined differences relative to non-Latino whites [19–24], very few studies have examined the role of acculturation in cessation behaviors. The research that has been published has relied on community or intervention studies based on nonprobability samples, and have produced inconsistent findings [17, 25, 26]. Moreover, ignored in the studies of acculturation and tobacco use and cessation is an examination of intermediate cessation behaviors, including quit attempts.

The lack of knowledge of the patterns of cessation behaviors among Latinos is cause for concern, particularly given the growth of this population in recent decades [27]. Additionally, Latinos are a heterogeneous population with varying health profiles. One striking characteristics is that roughly 40% is foreign born (approximately 30% excluding Puerto Rico) [28], a feature the tobacco industry has already recognized and incorporated it into their marketing practices [29]. Moreover, the USA Census Bureau estimates Latinos to be the fastest growing racial/ethnic group in the U.S.-projecting it will comprise about 25% of the total population by 2050, and net migration likely will play an important role in this growth [27, 30]. As such, it is crucial for tobacco behavior research to focus on all aspects of tobacco use behaviors and not just prevalence in this population.

The aim of this paper is to describe population level differences in cigarette smoking quit attempts among Latinos, with a focus on two measures often included in acculturation research-English language proficiency (ELP) and immigrant generation. In addition, we explore whether gender moderates the association between quit attempts and ELP or immigrant generation.

## 2. Methodology

### 2.1. Data Sources and Sampling Design

Data from the 2003 and 2006/07 Tobacco Use Supplement (TUS) to the Current Population Survey (CPS) were analyzed [31–34]. Details on the TUS-CPS methodology are described elsewhere [31–34]. Briefly, the TUS-CPS is a national survey of tobacco behaviors which employs a multistage probability sampling design [34]. The self-response rates ranged from 61% to 65.8% for the waves analyzed in this paper [32, 33]. The analysis was restricted to 4,589 adult (≥18 years of age) Latino current smokers and smokers who quit in the past 12 months (i.e., past year smokers). Proxy responses, persons under the age of 18, and those indicating they have never been regular smokers were excluded.

### 2.2. Variable Selection and Operationalization

#### 2.2.1. Outcome

The outcome was having made a quit attempt in the past 12 months (yes = 1, no = 0). Quit attempts were operationalized as, in the last 12 months, having stopped smoking for 1 day or longer because he/she was trying to quit smoking, having made a serious attempt to stop smoking because he/she was trying to quit even if he/she stopped for less than one day or having successfully stopped smoking.

#### 2.2.2. Focal Independent Variables

English language proficiency was dichotomized into “poor” versus “good” English ability. Respondents who conducted the interview in Spanish or another non-English language were assumed to have poor English language proficiency, while those who conducted the interview in English were assumed to have good English language proficiency. This is a proxy measure of language proficiency that has shown good agreement with the acculturation scale in the National Alcohol Survey (kappa =  .71) [35].

Immigrant generation was categorized to contrast first generation (foreign born individuals), second generation (USA born, with at least one foreign born parent), and third generation or higher (USA born, with 2 USA born parents; hereafter referred to as third generation).

#### 2.2.3. Control Variables

Control variables were selected based on previous empirical evidence in the tobacco control literature or the literature on Latino health. Sociodemographic control variables include education (less than high school, high school or GED, some college, or bachelor's degree or higher), annual household income (less than $25 K, $25 K to less than $50 K, $50 K to less than $75 K, and $75 K or more), and gender.

Age of smoking initiation and time to first cigarette in the morning were included to account for smoking behaviors and dependence. Age of initiation was categorized as “before 18,” “18 to 24,” and “25 years and older.” Time to first cigarette after waking was categorized as “less than 30 minutes,” “30 minutes or more,” and “varies.” Having received advice from a health care provider to stop smoking in the past 12 months was coded as “yes” versus “no”. Respondents without a health care visit in the past 12 months were regarded as not having received advice to stop smoking. Lastly, per capita tobacco control expenditures were included to control for the tobacco control context in which the respondents live. Following Farrelly et al. [36, 37] tobacco expenditures were computed to include 100% of the current year (i.e., year of data collection) per capita funding while discounting the three most previous years by 25% per year.

In addition to the variables described above, race, country of origin, and occupation type were included in the multivariable models if they met the criteria suggested by Hosmer and Lemeshow [38].

### 2.3. Statistical Analysis

Overall associations were estimated by fitting multivariable logit models, where log-odds of a quit attempt in the last 12 months was regressed on the focal independent variables and a set of control variables, with separate models for ELP and immigrant generation. Fitting separate models for ELP and immigrant generation was done to recognize that language proficiency is likely an intermediate variable between immigrant generation and the outcome, rather than the two focal independent variables being treated as confounders. Age was centered at the mean in the sample and cumulative per capita tobacco control funding was centered at the mean among states. Additionally, an interaction term for age of initiation and age was included to control for the differing effect age of initiation may have by age of respondents.

To assess whether gender moderates the focal relationships, product terms for gender and ELP and gender and immigrant generation were added to the respective models. The interactions were examined using the approach described by Norton et al. [39] and Ai and Norton [40]. However, the conclusion of the interaction analyses was consistent across the range of predicted probabilities. As such, for succinctness only the exponentiated logit coefficient for the interaction terms are presented and discussed in this paper.

The CPS is released with pre-imputed demographic information for some variables with missing values. The imputation methods for these variables are described elsewhere [34]. Categorical variables that were not pre-imputed were coded to include an “unknown” category and included in the models. One exception is for ELP, which had less than half a percent of observations missing.

All analyses were conducted in Stata 11 [41]. Sampling weights and balanced repeated replication weights (240 replicates) with Fay's adjustment factor were used to adjust the point and interval estimates for the complex survey design. Because the objective of the analysis was to describe population level patterns in quit attempts, 95% confidence intervals are presented and discussed rather than *P*-values. This allows for an assessment of the range of plausible values rather than using a testing approach for between group differences. Readers interested in assessing statistical significance can do so using the conservative approach of judging non-overlapping confidence intervals between groups [42].

## 3. Results

### 3.1. Sociodemographic Description

Table 1 provides univariate and bivariate descriptive statistics of the sociodemographic characteristics of Latino past year smokers in the study sample. Overall, about three out of four Latino past year smokers had good ELP and almost half were first generation immigrants. Nearly two-thirds of the respondents were male, and the mean age was 38 years. Less than ten percent had a bachelor's degree or higher while seven out of ten had either less than a high school education or a high school diploma or equivalent. About one in ten had an annual household income of $75,000 or more while over four in ten reported a household income of less than $25,000 annually. Almost three out of ten reported having a manual labor occupation, less than one in ten were unemployed, and two in ten were not in the labor force. Nearly six in ten reported Mexico as their Latino origin, while fifteen percent reported Puerto Rico, less than five percent reported Cuba. Finally, nine out of ten were white, while the rest were black or some other race.

Compared to Latinos with good ELP, those with poor ELP were more likely to be first generation immigrants, slightly older, male, have less than a high school education, have an annual household income less than $25,000, report Mexico as their country of origin, and identify themselves as white. Those with poor ELP were less likely than those with good ELP to have a management occupation and report Puerto Rico as their country of origin.

First generation immigrants were less likely than second and third generation immigrants to have good ELP, and have annual household income of at least $75,000. However, first generation immigrants were more likely than second and third generation immigrants to be male and have less than a high school education.

### 3.2. Smoking Characteristics of Latino Past Year Smokers

Table 2 presents univariate and bivariate descriptive statistics of the smoking characteristics of the Latino past year smoker population. Overall, just over half had made a quit attempt in the past year, about half began smoking regularly before their 18th birthday, two out of ten smoked their first cigarette within 30 minutes of waking in the morning, and three of ten reported having received advice to stop smoking from a health care provider in the past 12 months. Lastly, over half were current daily smokers, three in ten current someday smokers, and just over one in ten had stopped smoking in the 12 month period prior to the time of data collection.

Those with poor and good ELP were about equally likely to have made a quit attempt in the past 12 months. Second generation immigrants were more likely than third generation immigrants to have made a quit attempt. Those with poor ELP were less likely than those with good ELP to start smoking regularly before age 18, smoke their first cigarette within 30 minutes, and have received advice to stop smoking from a health care provider in the past 12 months. First generation immigrants were less likely than second and third generation immigrants to report beginning smoking regularly before 18 years of age, have their first cigarette within 30 minutes, and report having received advice to stop smoking from a health care provider in the last 12 months.

Those with poor ELP were slightly less likely to be daily smokers than those with good ELP but were equally likely to be former smokers. First generation immigrants were less likely than third generation immigrants to be daily smokers, while they were more likely than second and third generation immigrants to be someday smokers.

### 3.3. Multivariable Models

Table 3 presents unadjusted odds ratios (UOR) based on univariable logit regressions and adjusted odds ratios (AOR) with 95% confidence intervals (CI) of making a quit attempt in the past year by ELP, immigrant generation, and control variables. Overall, those with poor ELP were more likely to have made a quit attempt relative to those with good ELP (AOR = 1.22, CI: 1.02–1.46) after controlling for demographic and smoking characteristics. Similarly, first (AOR = 1.21, CI: 1.02–1.43) and second generation immigrants (AOR = 1.36, CI: 1.12–1.64) were more likely than third generation or higher immigrants to have made a quit attempt in the past 12 months.

Table 4 presents the models with interactions for gender and ELP and gender and immigrant generation. The AOR contrasting poor relative to good ELP is smaller by a factor of 0.78 (CI: 0.54–1.13) among males than among females. Similarly, the AOR contrasting 1st and 3rd generation immigrants is smaller by a factor of 0.86 (CI: 0.62–1.20) among males than among females, and the contrast between 2nd and 3rd generation immigrants is larger by a factor of 1.43 (CI: 0.96–2.13) among males than among females.

**Figure 1:**
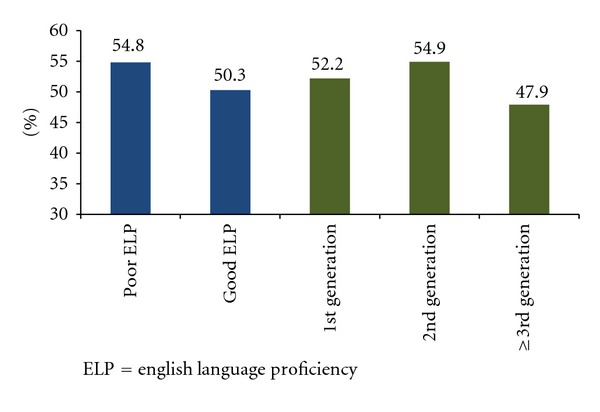
Predictive margins for making a quit attempt in the past 12 months by English language proficiency and immigrant generation among Latino past year smokers (*N* = 4, 589). ELP = English language proficiency.

Predictive margins for quit attempts by ELP and immigrant generation is presented in Figure 1. Latinos with good ELP (50.3%) had lower predictive margin of past 12 month quit attempt than Latinos with poor ELP (54.8%). First (52.2%) and second generation immigrants (54.9%) had higher predictive margins than third generation immigrant Latinos (47.9%).

## 4. Discussion

The present analyses found that Latinos with poor ELP and those of a more recent immigrant generation were more likely to have made a quit attempt. Interestingly, third generation Latino immigrants had similar predictive margin of quit attempt as the overall non-Latino white estimate of quit attempts (46.4%, data not shown in tables or figure). These findings are consistent with past research, which suggests that those with more exposure to USA culture adopt the prevailing tobacco behaviors, at least as compared to non-Latino whites, which is the comparison most often made in the tobacco control acculturation literature [1, 6, 8–10, 17]. Our findings demonstrate that disaggregating Latinos based on language and immigrant generation are warranted in future studies of smoking cessation attempts.

The analysis did not find reliable evidence that that gender moderates the associations between quit attempts and ELP or immigrant generation. However, it is noteworthy that the direction of the interaction observed in these data is consistent with much of the research in the cigarette smoking literature in that there appears to be a stronger acculturation effect among women than there is among men [2, 4, 5, 9, 12]. In contrast, our findings are inconsistent with an analysis by Castro et al., who found an acculturation effect only among men and not among women [17]. Comparative population data has shown that the smoking prevalence in many Latin American countries is much lower compared to the USA rates among women but much more similar among men, and that has been the case over the last several years [43–45]. As such, the larger acculturation effect among females might be expected for smoking simply because there is more room for overt behavior change. However, there is little comparative population data from Latin American countries for cessation behaviors, which to an extent hinders interpretation of the findings from the present analysis. To date, only Mexico, through the Global Adult Tobacco Survey (GATS), has comparative population level cessation behavior data available [46]. The GATS data show that 57% of female past year smokers in Mexico had made a quit attempt compared to 47% among men [46]. This compares to 49% for females and 44% for males in the non-Latino white sample of the TUS-CPS (data not shown in tables). If the Mexico data roughly extend to other Latin American countries, it is consistent with acculturation to see a stronger association among women than among men for quit attempts. As comparative population level data become more widely available as global tobacco control surveillance grows, this information should be incorporated in future analyses to aid in interpretation of other tobacco use and cessation behaviors.

### 4.1. Strength and Limitations

The major strength of this paper is that it examined a relationship that has not previously been reported in the published literature. Moreover, it did so using a large nationally representative dataset with rich data on current and past tobacco behaviors and sociodemographic information on the Latino population. However, our paper also has some limitations. First, due to the cross-sectional design we do not have the longitudinal data to support conclusions about changes in smoking behavior patterns over time. Second, the analysis was limited by the variables that were available in the TUS-CPS dataset. As such, variables such as smoking cessation cognitions and other psychological measurements that may be related to cessation could not be controlled for. Third, the concept of acculturation involves multiple aspects to identity formation and adaptation that are inherently dynamic and complex. We used measures commonly applied in the literature, but in recent years Latino health researchers have increasingly called attention to the need for theoretically based measures of acculturation and studies that begin to capture the full range of the Latino experience in the United States, particularly socioeconomic and racially/ethnically-based disadvantage [16, 47–49]. Lastly, the data in the TUS are self-reported and are subject to recall error, which may be differential with respect to current versus former smokers and ELP or immigrant generation.

## 5. Conclusion

In summary, our study adds to the growing literature on the heterogeneity of Latino health and extends prior work by presenting data on quit attempts. These findings underscore the need to disaggregate Latinos beyond racial/ethnic categories to identify subgroup differences relevant for smoking and smoking cessation behaviors in this population.

## Figures and Tables

**Table 1:** Sociodemographic characteristics of Latino past year smokers.

	Overall		English language proficiency				Generational status					
	(*N* = 4,589)		Good (*N* = 3,597)		Poor (*N* = 971)		1st (*N* = 2,016)		2nd (*N* = 879)		≥3rd (*N* = 1,694)	
	%	95% CI	%	95% CI	%	95% CI	%	95% CI	%	95% CI	%	95% CI
*ELP* ^†^												
Good	75.6	(73.8, 77.3)					52.2	(49.3, 55.0)	92.3	(89.2, 94.6)	98.5	(97.7, 99.0)
Poor	24.4	(22.7, 26.2)					47.8	(45.0, 50.7)	7.7	(5.4, 10.9)	1.5	(1.0, 2.3)
*Immigrant generation*												
1st generation	47.8	(45.9, 49.6)	33.0	(31.1, 35.0)	93.8	(92.0, 95.3)						
2nd generation	11.5	(10.4, 12.6)	13.9	(12.6, 15.3)	3.6	(2.5, 5.1)						
≥3rd generation	40.8	(38.9, 42.6)	53.1	(51.0, 55.3)	2.6	(1.7, 3.8)						
*Mean age in years*	38.0	(37.5, 38.4)	37.2	(36.7, 37.7)	40.3	(39.3, 41.2)	39.1	(38.5, 39.8)	35.8	(34.3, 37.3)	37.2	(36.6, 37.9)
*Gender*												
Male	64.8	(63.3, 66.2)	60.9	(59.1, 62.6)	76.9	(74.2, 79.5)	74.2	(72.4, 75.9)	57.1	(52.4, 61.7)	55.9	(53.6, 58.2)
Female	35.2	(33.8, 36.7)	39.1	(37.4, 41.9)	23.1	(20.5, 25.8)	25.8	(24.1, 27.6)	42.9	(38.3, 47.6)	44.1	(41.8, 46.5)
*Education*												
*<HS *	40.8	(38.9, 42.7)	32.7	(30.8, 34.8)	66.0	(62.5, 69.3)	53.7	(51.0, 56.4)	32.4	(27.8, 37.3)	28.0	(25.3, 30.8)
HS/GED	30.0	(28.4, 31.7)	33.3	(31.4, 35.3)	19.7	(16.9, 22.8)	23.7	(21.4, 26.1)	33.1	(28.6, 37.4)	36.5	(34.1, 39. 1)
Some college	21.7	(20.5, 23.0)	25.8	(24.3, 27.3)	8.7	(7.0, 10.7)	14.2	(12.7, 15.9)	28.9	(24.7, 33.5)	28.4	(26.2, 30.8)
BS^+^	7.5	(6.7, 8.5)	8.1	(7.2, 9.3)	5.7	(4.3, 7.5)	8.4	(7.2, 9.8)	5.6	(3.8, 8.3)	7.1	(5.9, 8.4)
Household income*												
<25 K	44.0	(42.2, 45.8)	40.7	(38.6, 42.7)	54.5	(50.8, 58.2)	46.1	(43.5, 48.7)	39.5	(35.7, 43.5)	43.7	(40.4, 47.1)
25 K to <50 K	33.1	(31.4, 34.9)	33.0	(30.1, 35.0)	33.5	(30.1, 37.1)	34.7	(32.2, 37.4)	33.1	(29.8, 36.6)	30.7	(27.8, 33.7)
50 K to <75 K	12.9	(11.6, 14.4)	14.2	(12.7, 15.9)	9.1	(6.8, 11.9)	11.3	(9.5, 13.5)	13.5	(11.0, 16.6)	14.9	(12.7, 17.3)
≥75 K	10.0	(9.0, 11.1)	12.2	(10.9, 13.7)	2.9	(1.7, 4.9)	7.8	(6.5, 9.3)	13.8	(11.3, 16.8)	10.8	(9.0, 12.9)
*Occupation type*												
Management	10.4	(9.4, 11.5)	12.7	(11.6, 13.9)	3.2	(2.2, 4.6)	7.5	(6.3, 9.0)	15.1	(11.8, 19.0)	12.4	(10.9, 14.2)
Service	15.4	(14.3, 16.6)	14.4	(13.1, 15.7)	18.6	(13.1, 21.4)	17.0	(15.3, 18.8)	12.0	(9.3, 15.4)	14.5	(12.9, 16.2)
Sales/Office	14.2	(13.1, 15.4)	16.8	(15.4, 18.3)	6.3	(4.9, 8.1)	9.3	(8.0, 10.8)	18.1	(14.3, 22.6)	18.9	(17.0, 21.0)
Manual labor	29.4	(27.9, 31.1)	25.0	(23.4, 26.8)	43.2	(39.8, 46.6)	39.0	(36.4, 41.7)	20.0	(16.5, 24.0)	20.8	(18.8, 23.0)
Unemployed	8.3	(7.4, 9.2)	8.8	(7.7, 10.1)	6.6	(5.1, 8.5)	6.8	(5.6, 8.1)	11.8	(8.8, 15.7)	9.0	(7.7, 10.5)
Not in labor force	22.3	(21.0, 23.7)	22.3	(20.7, 23.9)	22.1	(19.2, 25.3)	20.5	(18.5, 22.5)	23.0	(19.3, 27.2)	24.3	(22.2, 26.5)
*Race*												
White	90.6	(89.6, 91.5)	89.1	(87.9, 90.2)	95.3	(93.7, 96.6)	92.9	(91.5, 94.1)	89.6	(86.1, 92.4)	88.2	(86.4, 90.0)
Black	3.7	(3.1, 4.4)	4.1	(3.4, 4.9)	2.5	(1.6, 3.9)	3.3	(2.5, 4.3)	6.4	(4.3, 9.4)	3.4	(2.6, 4.6)
Other	5.7	(4.9, 6.5)	6.8	(5.9, 7.9)	2.2	(1.4, 3.4)	3.8	(2.9, 4.8)	4.0	(2.5, 6.3)	8.4	(7.0, 10.0)
*Country of origin*												
Mexico	59.2	(57.3, 61.0)	57.2	(55.1, 59.3)	65.3	(61.3, 69.1)	55.8	(53.1, 58.5)	42.9	(38.1, 47.7)	67.7	(64.8, 70.5)
Puerto Rico	15.1	(14.0, 16.3)	17.9	(16.5, 19.4)	6.4	(4.6, 8.8)	12.5	(11.0, 14.3)	39.0	(34.4, 43.6)	11.4	(9.8, 13.3)
Cuba	4.7	(4.0, 5.6)	3.1	(2.5, 3.9)	9.8	(7.6, 12.4)	7.1	(5.9, 8.6)	6.2	(4.3, 8.8)	1.6	(1.0, 2.5)
Other	21.0	(19.5, 22.5)	21.8	(20.1, 23.7)	18.6	(15.8, 21.8)	24.6	(22.5, 26.8)	12.1	(8.9, 16.2)	19.3	(17.0, 21.7)

*>5% missing; ^†^<1% missing; 95%CI = 95% confidence interval.

**Table 2:** Smoking characteristics of Latino past year smokers, overall and by English language proficiency and immigrant generation.

	Latino		Good ELP		Poor ELP		1st Generation		2nd Generation		≥3rd Generation	
	(*N* = 4, 589)		(*N* = 3, 597)		(*N* = 971)		(*N* = 2, 016)		(*N* = 879)		(*N* = 1, 694)	
	%	95% CI	%	95% CI	%	95% CI	%	95% CI	%	95% CI	%	95% CI
*Quit attempt*, *past 12 months *	51.3	(49.6, 53.0)	51.3	(49.4, 53.3)	51.4	(47.8, 55.0)	51.0	(48.5, 53.5)	57.1	(53.5, 60.7)	48.2	(45.4, 51.1)
*Age smoked regularly* ^†^												
<18	51.9	(50.3, 53.5)	53.3	(51.5, 55.1)	47.5	(43.8, 51.2)	48.0	(45.5, 50.6)	56.3	(53.4, 60.1)	54.8	(52.4, 57.1)
18–24 years	38.8	(37.1, 40.4)	37.9	(36.1, 39.8)	41.4	(37.7, 45.2)	41.2	(38.7, 43.6)	37.6	(33.8, 41.5)	36.0	(33.4, 38.7)
25 years or older	9.4	(8.5, 10.3)	8.8	(7.8, 9.8)	11.1	(9.0, 13.7)	10.8	(9.3, 12.6)	6.1	(4.7, 8.1)	9.2	(7.6, 11.1)
Time to first cigarette*												
<30 minutes	20.7	(19.3, 22.2)	22.8	(21.2, 24.4)	14.4	(12.1, 17.1)	16.9	(14.9, 19.1)	22.4	(19.3, 26.0)	25.3	(22.9, 28.0)
≥30 minutes	76.1	(74.6, 77.6)	74.8	(73.2, 76.7)	79.8	(76.5, 82.6)	78.9	(76.5, 81.2)	75.1	(71.4, 78.5)	72.6	(69.9, 75.2)
Varies	3.2	(2.5, 4.0)	2.3	(1.7, 3.1)	5.8	(4.2, 8.0)	4.2	(3.2, 5.5)	2.5	(1.4, 4.2)	2.1	(1.4, 3.1)
*Advice to quit* ^‡^												
Yes	32.5	(30.9, 34.1)	35.9	(34.1, 37.8)	21.7	(19.0, 24.7)	27.4	(25.1, 29.7)	38.3	(35.0, 41.8)	36.4	(33.6, 39.2)
No	67.6	(65.9, 69.2)	64.1	(62.2, 65.9)	78.3	(75.3, 81.0)	72.6	(70.3, 74.9)	61.7	(58.2, 65.1)	63.7	(60.9, 66.4)
*Smoking status at time of survey*												
Current daily	57.0	(55.3, 58.7)	58.7	(56.8, 60.6)	51.8	(48.3, 55.2)	53.9	(51.5, 56.3)	57.4	(53.3, 61.3)	61.4	(58.5, 64.3)
Current someday	30.4	(28.8, 32.0)	28.7	(26.9, 30.5)	36.0	(32.9, 39.2)	33.5	(31.3, 35.8)	27.8	(24.4, 31.4)	27.5	(24.9, 30.2)
Quit in past year	12.6	(11.4, 13.8)	12.6	(11.3, 14.1)	12.2	(10.2, 14.7)	12.6	(11.0, 14.4)	14.9	(12.4, 17.8)	11.1	(9.3, 13.1)

95%CI = 95% confidence interval.

*>5% missing; ^†^>1% missing; ^‡^<1% missing.

**Table 3:** Odds ratios for making a quit attempt in the past 12 months, by ELP, immigrant generation, and covariates (*N* = 4, 589).

	Univariable logit		Multivariable model:		Multivariable model:	
	regressions		English language proficiency		immigrant generation	
	UOR	95%CI	AOR	95%CI	AOR	95%CI
*ELP*						
Poor	1.00	(0.85, 1.18)	1.22	(1.02, 1.46)		
Good	1.00	Referent	1.00	Referent		
*Immigrant generation*						
1st generation	1.12	(0.96, 1.30)			1.21	(1.02, 1.43)
2nd generation	1.43	(1.20, 1.71)			1.36	(1.12, 1.64)
≥3rd generation	1.00	Referent			1.00	Referent
*Gender*						
Female	1.00	Referent	1.00	Referent	1.00	Referent
Male	0.87	(0.75, 1.00)	0.94	(0.81, 1.09)	0.94	(0.81, 1.09)
*Per cap tob control exp*	1.01	(1.00, 1.03)	1.02	(1.00, 1.03)	1.02	(1.00, 1.03)
*Race*						
White	1.00	Referent	1.00	Referent	1.00	Referent
Black	1.40	(0.96, 2.04)	1.39	(0.96, 2.03)	1.32	(0.90, 1.94)
Other	0.90	(0.68, 1.18)	0.80	(0.61, 1.06)	0.82	(0.62, 1.08)
*Age (centered)*						
Age	0.98	(0.98, 0.99)	0.98	(0.97, 0.99)	0.98	(0.97, 0.99)
*Age of initiation*						
<18	1.00	Referent	1.00	Referent	1.00	Referent
18–24	1.12	(0.97, 1.28)	1.12	(0.94, 1.33)	1.13	(0.95, 1.34)
25+	1.05	(0.82, 1.34)	1.26	(0.96, 1.67)	1.27	(0.96, 1.67)
Unknown	0.41	(0.23, 0.72)	0.32	(0.15, 0.68)	0.36	(0.18, 0.73)
Age*age of initiation						
18–24 *age	1.00	(0.99, 1.01)	1.00	(0.99, 1.01)	1.00	(0.99, 1.01)
25+ *Age	0.99	(0.97, 1.01)	0.99	(0.98, 1.01)	0.99	(0.97, 1.01)
Unknown *age	0.99	(0.95, 1.03)	0.99	(0.93, 1.04)	0.99	(0.95, 1.04)
*Education*						
*<*High school	0.83	(0.70, 0.99)	0.88	(0.74, 1.05)	0.90	(0.75, 1.07)
High school/GED	1.00	Referent	1.00	Referent	1.00	Referent
Some college	1.10	(0.91, 1.32)	1.04	(0.86, 1.25)	1.02	(0.85, 1.23)
≥Bachelor	1.11	(0.85, 1.46)	1.01	(0.76, 1.33)	0.99	(0.75, 1.31)
*Household income*						
<25 K	1.00	Referent	1.00	Referent	1.00	Referent
25 K to <50 K	1.03	(0.87, 1.22)	0.95	(0.79, 1.14)	0.94	(0.78, 1.12)
50 K to <75 K	1.06	(0.84, 1.35)	0.88	(0.70, 1.12)	0.87	(0.69, 1.11)
≥75 K	1.41	(1.07, 1.86)	1.24	(0.93, 1.65)	1.18	(0.89, 1.57)
Unknown	0.83	(0.64, 1.09)	0.76	(0.56, 1.02)	0.76	(0.56, 1.01)
Time to first cigarette						
<30 minutes	1.00	Referent	1.00	Referent	1.00	Referent
≥30 minutes	1.25	(1.06, 1.47)	1.20	(1.01, 1.42)	1.20	(1.01, 1.44)
Varies	1.11	(0.72, 1.71)	1.15	(0.72, 1.83)	1.22	(0.77, 1.93)
Unknown	17.00	(9.55, 30.18)	17.55	(10.11, 30.48)	17.93	(10.35, 31.04)
Advice from HCP						
Yes	1.41	(1.23, 1.62)	1.58	(1.36, 1.83)	1.55	(1.34, 1.80)
No	1.00	Referent	1.00	Referent	1.00	Referent
Unknown	0.85	(0.39, 1.86)	0.65	(0.22, 1.91)	0.64	(0.25, 1.69)

Mean residual goodness of fit statistic			*F*_(9,231)_ = 1.01, *P* > .05		*F*_(9,231)_ = 1.80, *P* > .05	

AOR = adjusted odds ratio; UOR = unadjusted odds ratio; 95%CI = 95% confidence interval.

ELP = English language proficiency; HCP = health care provider.

**Table 4:** Adjusted odds ratios for making a quit attempt in the past 12 months by ELP, immigrant generation, gender, and interactions for ELP ∗ gender and immigrant generation ∗ gender (*N* = 4, 589).

	Multivariable model: English language proficiency^a^		Multivariable model: immigrant generation^a^	
	AOR	95%CI	AOR	95%CI
*ELP*				
Poor	1.47	(1.08, 2.00)		
Good	1.00	Referent		
*Immigrant generation*				
1st generation			1.35	(1.04, 1.76)
2nd generation			1.10	(0.84, 1.44)
≥3rd generation			1.00	Referent
*Gender*				
Female	1.00	Referent	1.00	Referent
Male	0.99	(0.84, 1.17)	0.92	(0.73, 1.16)
*Interactions*				
ELP ∗ gender	0.78	(0.54, 1.13)		
1st generation ∗ gender			0.86	(0.62, 1.20)
2nd generation ∗ gender			1.43	(0.96, 2.13)

^a^Controls for cumulative per capita tobacco control expenditures, race, education, income, time to first cigarette, cessation advice from healthcare provider, age, age of initiation, interaction of age and age of initiation.

AOR = adjusted odds ratio.

95%CI = 95% confidence interval.
